# Automated Detection of Vessel Abnormalities on Fluorescein Angiogram in Malarial Retinopathy

**DOI:** 10.1038/srep11154

**Published:** 2015-06-08

**Authors:** Yitian Zhao, Ian J. C. MacCormick, David G. Parry, Nicholas A. V. Beare, Simon P. Harding, Yalin Zheng

**Affiliations:** 1 School of Mechatronical Engineering, Beijing Institute of Technology, Beijing, China; 2 Department of Eye and Vision Science, University of Liverpool, Liverpool, UK; 3 Malawi-Liverpool-Wellcome Trust Clinical Research Programme, Blantyre, Malawi; 4 St. Pauls Eye Unit, Royal Liverpool University Hospital, Liverpool, UK

## Abstract

The detection and assessment of intravascular filling defects is important, because they may represent a process central to cerebral malaria pathogenesis: neurovascular sequestration. We have developed and validated a framework that can automatically detect intravascular filling defects in fluorescein angiogram images. It first employs a state-of-the-art segmentation approach to extract the vessels from images and then divide them into individual segments by geometrical analysis. A feature vector based on the intensity and shape of saliency maps is generated to represent the level of abnormality of each vessel segment. An AdaBoost classifier with weighted cost coefficient is trained to classify the vessel segments into normal and abnormal categories. To demonstrate its effectiveness, we apply this framework to 6,358 vessel segments in images from 10 patients with malarial retinopathy. The test sensitivity, specificity, accuracy, and area under curve (AUC) are 74.7%, 73.5%, 74.1% and 74.2% respectively when compared to the reference standard of human expert manual annotations. This performance is comparable to the agreement that we find between human observers of intravascular filling defects. Our method will be a powerful new tool for studying malarial retinopathy.

Cerebral malaria (CM) is a major cause of death and disability, especially in children in sub-Saharan Africa. CM is characterised by sequestration of parasitised erythrocytes in cerebral vessels^1^, but despite much research the mechanisms by which the intravascular malaria parasite causes coma and death remain unclear^2^,^3^. Malarial retinopathy (MR) has been identified as an important clinical sign in the diagnosis and prognosis of cerebral malaria^4^. The retina and brain are affected in similar ways in CM, and so the photographic features of MR are likely to give further valuable information about CM disease process, diagnosis, treatment and prognosis^2^.

Intravascular filling defects (IVFD) are a feature of MR that can be observed in fluorescein angiogram (FA) images. IVFD may represent sequestration of parasitised erythrocytes in the microvasculature^5^. Sequestration is the pathological hallmark of cerebral malaria^6^,^7^, but as yet, it has only been possible to quantify it histopathologically at post mortem. IVFD can be seen in large and small venules, arterioles and capillaries, but appear to be most prominent in venules. As shown in Fig. 1, the appearance ranges from mottling and slight irregularities of the vessel wall, to more obvious lesions that look as if small bites have been taken from the vessel^4^,^8^. Cerebral and retinal sequestration is always seen in fatal cases of CM with MR^2^,^9^, and the histopathological appearance of sequestration is similar to IVFD^2^,^10^. Moreover, IVFD often resolve the day after treatment with anti-malarial drugs is started (personal observation). This is consistent with resolution of sequestration and clinical recovery. It is plausible that IVFD represent this fundamental pathological process, and this lesion merits further investigation.

Previous studies of FA in severe malaria were based on semi-quantitative data based on human observation^11^. Manual grading is often time consuming, is subject to observer variations^12^, and may not adequately capture important details such as the precise extent or location of lesions. By overcoming some of the limitations of manual grading, automated detection of IVFD may help to illuminate CM disease mechanisms. Unlike retinal haemorrhages, and capillary non-perfusion^13^, to the best of our knowledge automated quantification of IVFD has not yet been attempted. We address this by presenting a framework for automated detection of IVFD, with the aim of quantifying an under-researched retinal feature that has plausible links to the fundamental disease process involved in cerebral malaria.

Automated vessel analysis is an active research area in the field of medical imaging^14^. The primary effort has been focused on automated vessel segmentation, as evidenced by extensive reviews^15^,^16^, and quantitative measurements of vessel geometry such as arteriovenous ratio (AVR), tortuosity, and fractal number^14^. There are few works on automatic vasculature analysis in FA^17^,^18^, and within this literature, the detection of discrete vessel abnormalities involving specific sections of the vessel wall has received little, if any, attention. Only one study addresses the related objective of detecting arteriolar narrowing in color fundus photography^19^. In their work^19^, a density analysis method is first used to detect the vessels, then connectivity analysis is performed to establish vessel trees, and finally arterioles are separated from venules by analysing vessel colour and width so as to assess arteriolar narrowing. This method had a sensitivity of about 75%.

We propose a new framework for automated detection of IVFD. Essentially we have formulated the problem in terms of image classification, where the objective is to train a classifier to determine if a vessel segment is normal or not based on a set of features that represent each segment. Throughout this paper, a vessel segment is defined as a connected segment of the detected vasculature between junctions or bifurcations, or a segment containing only one endpoint. The proposed framework will address three major challenges: 1) Accurate, efficient and reliable detection of vessels; 2) The process of deriving the features that are most discriminative and able to separate normal and abnormal vessels. 3) A classifier with good performance has to be identified and trained properly.

Our framework includes graph cut-based vessel segmentation, vessel geometry analysis, saliency map generation, and ensemble classification by AdaBoost (details of these technical components are described in the methods, below).

*Saliency* is a predictor of object regions which attract human attention. It indicates the relative importance of visual features and is closely related to characteristics of human perception and processing of visual stimuli^20^,^21^,^22^. Saliency originates from visual uniqueness, unpredictability, rarity, or surprise, and is often attributed to variations in image attributes like colour, gradient, edges, and boundaries^23^. Saliency in 2D images is the perceptual quality that makes an object, person, or pixel stand out relative to its neighbours, and that captures our attention^22^. Estimated saliency maps are widely used in many computer vision applications including object of interest image segmentation^24^, object recognition^25^, and so on. A pixel is salient if its appearance is unusual, considering the context of neighbouring pixels - one always looks at a pixel within its surrounding patch rather than simply observing a pixel in isolation. We define saliency in terms of information content: a key-point corresponds to a particular image location within a structure with a low probability of occurrence (i.e. high information content). Many saliency detection approaches for 2D images exist. They have a similar structure, computing several features in parallel and then fusing their values in a representation which is usually called a *saliency map*. The most general model of saliency detection is described by Itti and Koch^21^. Other existing saliency detection methods for feature determination can be divided into four classes: pixel-based methods^21^,^26^,^27^,^28^,^29^,^30^; region-based methods^22^,^23^,^31^; frequency-based methods^32^,^33^,^34^,^35^; parameter learning-based methods^36^,^37^,^38^.

In the case of IVFD, there is a contrast between the normally smooth vessel wall and individual discrete lesions that appear to protrude into the vessel lumen (Fig. 1(a)). These lesions may be defined as salient regions. Similarly, in the vessels affected by IVFD, some sections of the diameters or curvatures of vessel walls may be significantly different from neighbouring vessels or even other segments of the same vessel (Fig. 1(b)), such vessel edges may also be determined as salient features. These observations prompted us to use vessel intensity and shape saliency maps, and combine them to generate a combined saliency map.

## Results

In this section we describe the dataset used, evaluation metrics, experiments performed to evaluate the effects of various parameters, and the experimental results.

### Dataset

Our automated framework was evaluated against a dataset containing 6,358 vessel segments (3,033 abnormal segments) from 10 retinal FA images with a size of 3008 × 1960 pixels. These images were taken in the children with CM admitted to the Malaria Research Project Ward, Department of Paediatrics, Queen Elizabeth Central Hospital, Blantyre, Malawi. All subjects had signs of MR on admission. Ethical approval for retinal examination and imaging was given by committees in Blantyre and at collaborating institutions. Consent was given by the parents/guardians of subjects before examination and imaging. The tenets of the Declaration of Helsinki were adhered to. 50-degree images were taken after pupil dilation with Tropicamide 1% and Phenylephrine 2.5%, using a Topcon 50-EX optical unit (Topcon, Tokyo, Japan) and Nikon E1-H digital camera. Manual annotation of IVFD is extremely time consuming even aided by computer programs, it takes over an hour per image. Therefore, only 10 representative cases were selected for the evaluation of IVFD detection. We intentionally chose images that display a range of IVFD severity to create this dataset. This selection was made by ophthalmologists and professional graders who have been leading concurrent development of a protocol for manual grading of IVFD and other retinal features in cerebral malaria. Although the number of subjects is relatively small, we feel that these images represent a fair range of this spectrum.

Human expert graders used a systematic approach to label vessels as abnormal or normal in terms of IVFDs aided by an in-house Matlab program version 2013a (Mathworks, Natick, CA). During the process, the original and an overlay image of the original with centrelines of vessels highlighted in yellow were displayed side by side. Observers were asked to select abnormal and normal vessel segments in turn by clicking on the vessel segment of interest. The selected abnormal segments were then highlighted in red while normal ones in green. In order to assess the detection performance of the framework on vessel segments with different diameters, the observers were asked to look at the peri-capillary vessels, small vessels or large vessels separately. Following our in-house FA grading workbook, we define capillaries as the smallest vessels visible on a well-focussed angiogram. A post-capillary venule is formed by the confluence of two or more capillaries, and extends up to the point where it is joined by a second post-capillary venule or other larger venular segment. Small venules are defined as any section of vein between the edge of the post-capillary venule complex up to the point of confluence with another vessel of similar or larger calibre. Large venules extend from the point where two small venules converge to the edge of the optic disc.

Three experienced observers in grading MR images were involved in the grading. A professional grader (DGP) and an ophthalmologist (IJCM) labelled the vessels using the same software and following the same guidelines in a masked pattern. The grading results by DGP were reviewed together by a senior ophthalmologist familiar with IVFDs (SPH) and the consensus between them was used as the final reference standard. When human graders were uncertain whether IVFDs were present or absent, vessels were left unlabelled and are not analyzed in this study.

### Evaluation Metrics

Four commonly-used metrics were employed to evaluate the performance of the program in terms of vessel segment: sensitivity, specificity, accuracy, and the area under a receiver operating characteristic curve *AUC*. Sensitivity is a measure of effectiveness in identifying abnormal vessel segments while specificity performs the same function for normal vessel segments. Accuracy indicates the overall classification performance. *AUC* has the ability to reflect the trade-offs between the sensitivity and specificity in particular in the case of imbalanced data classification. These metrics are defined as follows:


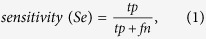



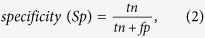



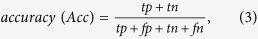



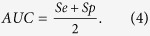


where *tp*, *tn*, *fp* and *fn* indicate the true positive (the number of correctly identified abnormal vessel segments), true negative (the number of correctly identified normal vessel segments), false positive (the number of incorrectly identified abnormal vessel segments), and false negative(the number or incorrectly identified normal vessel segments), respectively. In particular *AUC* is calculated as suggested by Hong *et al.*^39^. An AUC of 1.0 means that the classifier distinguishes class examples perfectly.

### Experiment Settings

The 10 images in the dataset were randomly separated into a training set (8 images) and testing set (2 images). The training set was used to train and validate models while the testing set for evaluating the performance of the final model. An image-wise partition strategy was chosen in order to avoid possible overfitting, which could be introduced by a segment-wise partition strategy. With a segment-wise partition strategy, a classifier trained and tested on vessel segments from the same images may provide surprisingly good results on the training images, but perform poorly on new images. We applied repeated leave-one-out cross validation (LOOCV) to the training set for parameter optimization (or model selection)^40^. In brief, of the 8 images in the training set, 7 images were used to train a model while the remaining image was retained as the validation data for testing the model trained. The process was repeated 8 times with each single portion (image) used exactly once as the validation data. The LOOCV was then repeated five times on different random splits of the dataset, and the mean values of sensitivity, specificity, accuracy and *AUC* were used for comparisons of different parameter settings. The range tested for the number of trees was 500, 1000, 2000, 5000 and 10,000 while the range for the cost coefficient was 2 to 8 with an interval of 2. The ‘optimal’ values of the class weights and number of trees found from the repeated LOOCV were used to train the whole training set to obtain the final model. The performance of the final model was determined by applying it to the testing set. Sub-analysis on the performance of the final model for detection of vessel segments at different types was also performed.

### Experimental Results

**Figure 2:**
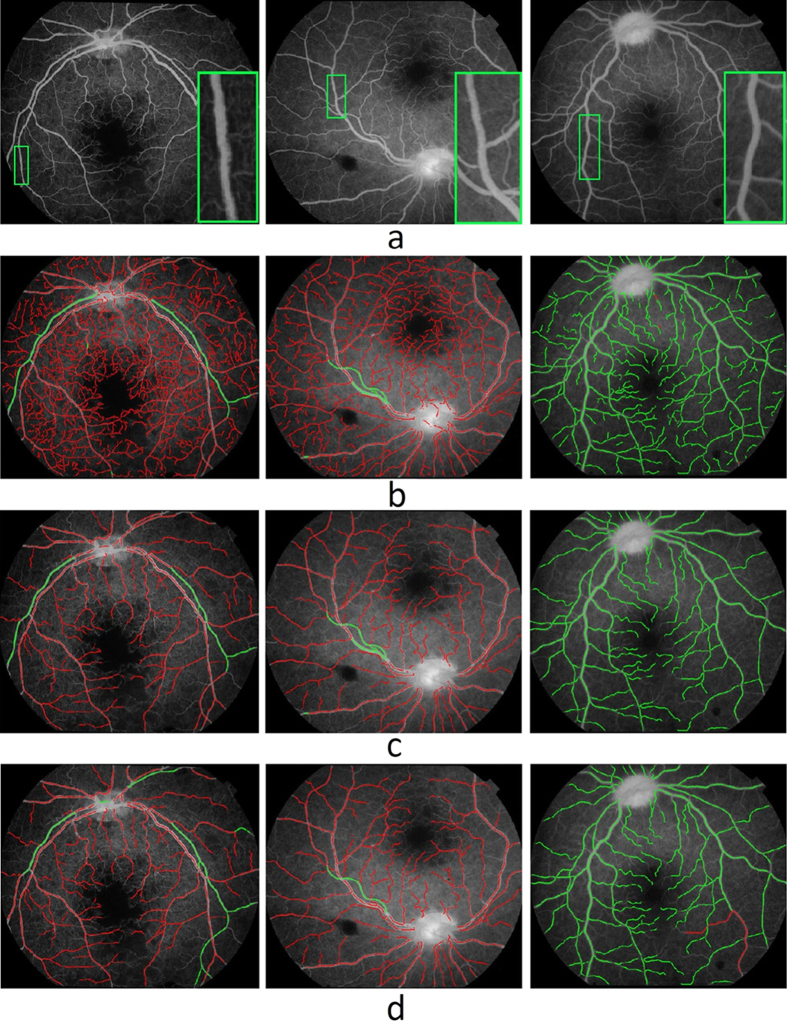
Comparison of abnormal vessel detection between the automated framework and manual annotations (abnormal vessels are highlighted in red and normal vessels in green). (**a**) Three example fluorescein angiography images. The inset in each image shows the zoom-in view of the region enclosed by the green box within that image. (**b**) Detection results on all the segmented vessels by the framework. (**c**) Detection results on the gradable vessels only by the human observers. (**d**) Manual annotations from a consensus of two observers was used as the reference standard.

Figure 2 shows the results of the proposed automated abnormal vessel detection framework on 3 FA images where the normal vessels are illustrated in green colour whilst the abnormal vessels are in red colour. As we can see from Fig. 2(b), our method has classified all the vessel segments segmented by our vessel segmentation method into normal and abnormal segments respectively. However, there were a number of thin vessels that were ungradable for the human observers due to poor contrast. In this work, only the vessels labelled by human observers were considered for the purpose of comparison. Comparing results from our automated method (Fig. 2(c)) with those of human observer’s (Fig. 2(d)), it can be seen that the results are visually very similar either in the case of lots of abnormal vessels contained images (Fig. 2 left and middle column) or fewer abnormal contained image (Fig. 2 right column).

**Figure 3:**
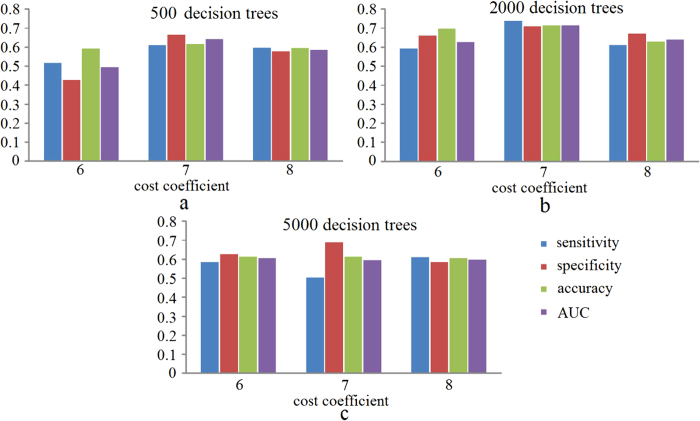
Evaluation results in terms of sensitivity, specificity, accuracy, and area under the curve (*AUC*), under different combinations of decision trees and cost coefficients. (**a**) Results using 500 trees; (**b**) Results using 2000 trees; (**c**) Results using 5000 trees. On each plot, from left to right are the results with cost coefficient of 6, 7, and 8 respectively.

Figure 3 shows the classification performance for different values of the cost coefficient and different numbers of decision trees. It is clear that the classifier with 2000 trees and a cost coefficient of 8 was most effective in the detection of IVFD. With this set of parameters we were able to train a final model and apply it to the test set to obtain the evaluation results of the proposed method with the consensus annotations. Table 1 shows that the evaluation results in terms of sensitivity, specificity, accuracy, and *AUC* are 0.747, 0.735, 0.741, and 0.742, respectively. In addition, The overall inter-observer agreement for IJCM and DGP was found to be *κ* = 0.424 (*p* < 0.001) implying good agreement. The *κ* value for the framework and DGP is 0.555 (*p* < 0.001).

**Figure 4:**
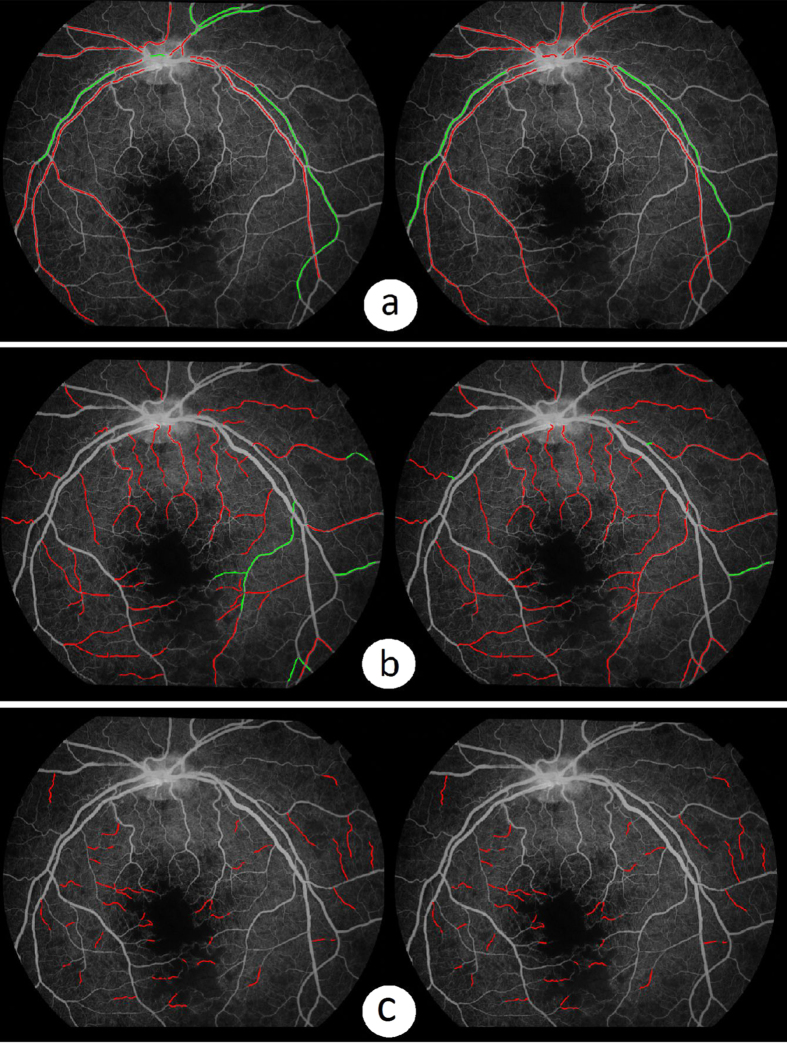
Illustration of the detection performance of the framework on vessels with different sizes (Left column: Manual annotations. Right column: Automated annotations). From top to bottom, (**a**) Results on large vessels. (**b**) Results on small vessels. (**c**) Results on peri-capillary vessels.

In order to provide clinicians with more information about abnormalities in vessel segments, we also evaluated the performance on large, small and peri-capillary vessels separately. Figure 4 shows the results on one image by the program and the expert annotation side by side and Fig. 4(a–c) show the results on large, small and peri-capillary vessels respectively. The results for these three vessel types in terms of sensitivity, specificity, accuracy, and *AUC* were also presented in Table 1. Overall, the proposed abnormal vessel detection for the vessels from small vessel has the highest performance, which achieve sensitivity of 0.765, specificity of 0.782, accuracy of 0.751, and *AUC* of 0.776.

### Discussion and Conclusions

We have developed a novel abnormal vessel detection framework to identify IVFD – a neurovascular sign that may represent an important part of CM pathogenesis. The framework comprises four major components: vessel segmentation, analysis of vessel geometry, salient feature generation, and vessel classification. Our evaluation of this framework yielded results that are comparable to expert human observers. While much work has been done to develop tools to measure retinal vessel geometry, to the best of our knowledge this is the first report of automated analysis of discrete retinal vessel abnormalities.

Our method demonstrated satisfactory overall performance: sensitivity of 74.7%, specificity of 73.5%, and accuracy of 74.1%. In terms of vessel type-wise analysis, the framework achieved a sensitivity of 76.4%, specificity of 79.1%, and accuracy of 75.9% on small vessel. These results are consistent with the fact that there are relatively few large vessels, compared to smaller vessels. Unfortunately peri-capillary vessels were not typically photographed with sufficient quality for analysis to be accurate.

These promising results largely rely on our novel adaptation of the concept of salient features to the field of medical image analysis. In psychological terms, saliency is a predictor of visual object regions that attract human attention. Saliency indicates the relative importance of components of our visual world, and is closely involved in perception and processing of visual stimuli. In computational terms, saliency refers to a region or object that stands out from its neighbours or background. In this paper, we represented IVFD as salient regions on the background of the retinal image. IVFD can be thought of as minute vessel regions that have different diameter, curvature, or contrast to neighbouring regions. These features of IVFD are in line with the definition of saliency in computer vision field: the salient region is one that is significantly different from nearby regions in terms of contrast or shape.

Another highlight of our approach is the use of weighted ensemble classification method to deal with imbalanced data. This is very important as the proportion of abnormal to normal vessel segments in a retinal image is often skewed. A weighted classification strategy appears to be an appropriate way to penalize misclassification errors for each class differently. Furthermore, an ensemble classification technique will usually provide better performance compared to single classifiers. We chose weighted AdaBoost for this specific application because of its simplicity, efficiency and robustness against potential problem of overfitting. Other classification methods, such as weighted-SVM^41^, could also be used.

Automated analysis of retinal images is an important objective in medical research. The main emphasis has been on analysis of colour fundus photographs rather than FA, and on quantifying vessel geometry rather than identifying particular vessel segments affected by focal lesions. As a result the problem of detecting discrete vessel abnormalities is relatively unexplored. Achieving high performance in automated lesion detection is a challenging task. In our experience, there are many different factors that could compromise performance. First of all, there is often a very large variation in brightness, contrast, and artefact across images. This makes it difficult to have universal criteria to define the abnormalities. Secondly, IVFD can be difficult to grade, even for expert human graders. It is possible that an automated technique such as ours might provide more accurate detection than the current human expert reference standard.

Development of this framework is motivated by medical demands for a tool to measure the number of abnormal vessels in retinal FA images, and our method should allow better estimation of associations between MR and clinical outcome in patients with CM. This work is ongoing. The flexibility of this framework suggests it might be suitable for detecting abnormal vessel segments in other retinal or neurovascular diseases that involve discrete vascular lesions.

In conclusion, we have proposed and evaluated an innovative abnormal vessel detection framework to support the study of malaria retinopathy, and our experimental results have demonstrated its effectiveness. It has potential to be further developed as a useful tool for fast accurate and objective assessment for a range of retinal diseases.

## Methods

In this section the proposed automated IVFD detection framework is described in detail.

### Vessel Segmentation

**Figure 5:**
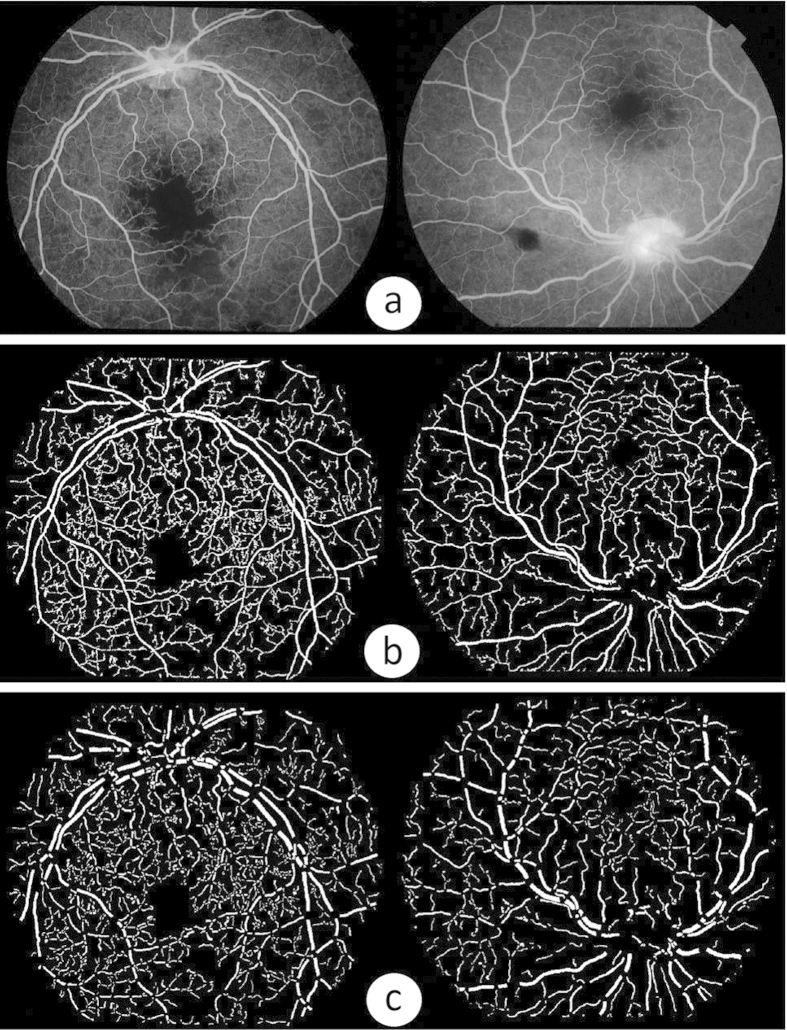
Illustration of vessel segmentation and geometric analysis results. (**a**) Two example fluorescein angiography images. (**b**) Vessel segmentation results. (**c**) Vessel segments after removing branch pixels from images in the second row. In (**b**) and (**c**) pixels in white denote vessels.

The automated detection of blood vessels is a prerequisite in the development of automated system for the analysis of vessels. For this work, we adopted a state-of-the-art segmentation technique for its good accuracy and efficiency^42^. This technique is built on local phase enhancement and graph cut method. Local phase-based vessel enhancement is employed to enhance vessel-like structures in an image to form a ‘vesselness map’. As suggested by the name, this filter uses local phase information in the image to enhance vessel-like structures. Compared to the conventional intensity-based filters, this filter is invariant to intensity inhomogeneity within the image and also capable of producing more accurate enhancement results for vessels with different widths, even at the bifurcations or end of vessels. The vessels are segmented by applying a graph-cut based Chan-Vese (CV) model to the vesselness map for its computational efficiency. This model^43^ as a region-based active contour model, segments the image into two regions (objects and background) by minimizing an energy for smooth boundary and low intra-region intensity variance. In this work we use the optimal parameter values as suggested by the original paper. In particular, for the graph-cut segmentation model, initialisation is achieved automatically by applying a threshold with an empirically chosen value of 0.5 to the vesselness map (afterwards ‘1’ denotes vessel pixel while ‘0’ background). Effects of different threshold values have been evaluated and it seems that the final results are not sensitive to it. Figure 5(a) shows two original example FA images, and their segmentation results are illustrated on Fig. 5(b).

### Geometric Analysis of Vessels

Following the vessel segmentation step, geometrical analysis of the segmented vessels is performed in order to split the vasculature into individual segments for further processing. The morphological thinning algorithm is first applied to the segmented vessel trees in order to estimate the centre line and diameter of vessel segments: the exterior pixels from the segmented vessels are removed iteratively by using the thinning algorithm, and obtaining a new binary image containing connected lines of ‘on’ pixels locating along the vessel centres. The centerlines are refined by using a least-squared cubic spline technique in order to obtain smoother trajectories^44^. Branch points (>2 neighbours) are removed so as to divide the centrelines of the vascular tree into individual portions where each portion corresponds a vessel segment. Segments with a short centreline length (<10 pixels) are eliminated to improve the speed of the later processing. Guided from the centerline location of each segment, individual vessel segments will be isolated from the original segmentation result by removing the branch points and their neighbour pixels. Figure 5(c) demonstrates the vessel segments produced after removing the branch pixels and pixels around them of Fig. 5(b).

The vessel diameters of each segment are estimated by using the distance transform of the inverted binary segmented image as suggested by^45^. It uses the Euclidean distance of each vessel pixel from the closest non-vessel pixel, and thus, doubling the maximum values of the distance transform along the thinned centerlines provides an estimate of the diameter of every vessel segment at its widest point. Bankhead *et al.* has demonstrated that this method can provide good width estimation results at locations in the middle of vessel segments^45^. It seems to us that this method may suffer at the two ends of vessel segments due to the complex geometry. In order to avoid this problem, only diameters at locations 5 pixels away from branch (or end) pixels are considered for the subsequent analysis. A segment will be removed if its centreline contains fewer pixels than its estimated mean diameter. After this process, each segment will be indexed for subsequent analysis.

### Feature Generation

To classify the vessel segments detected in the previous step as normal or abnormal, a set of features need to be derived to represent each vessel segment so as to form an input vector for the classifier to be used. In this work, for each segment, a total of 21 features including intensity and shape saliency maps are generated.

### Intensity-based Vessel Saliency

Let *w*(*x*) ∈ *V* to be the viable local representation as a patch that represents pixel *x*, and *V* indicates all the vessel segments. The average vessel diameter of our dataset is around 5 pixels, so the size of the patch is set as 3 × 3 in this work, where *x* is the centre of the patch. The patches can be seen as samples of a multivariate probability function (PDF). A number of methods to estimate an unknown multivariate PDF with a sufficient number of samples have been introduced in the literature. The kernel density estimator (KDE) is chosen in this paper. The KDE is appropriate since it is non-parametric, which will allow to estimate any PDF. Therefore, the probability of a patch *w*(*y*) can be defined as


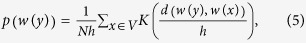


where *d* is a distance function that will be discussed later, *K* is a kernel, *h* is a smoothing parameter, and *N* represents the number of pixels. KDE method is capable to blur the contribution of each sample *x* by spreading it to a certain area in vessel segments with a certain shape^46^, which is defined by *K*. The multivariate distribution will have higher probability if the patches are in dense areas. From our experience, the most commonly used and the most appropriate kernel is Gaussian function with zero mean and standard deviation *σ*_*k*_. Using a Gaussian kernel, Eq. (5) is rewritten as


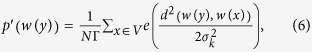


The estimated probabilities are taken from an actual PDF by setting a proper constant Γ. *σ* = 0.2 is chose to substitute *h*. After determining the probability of the patches, the intensity-based saliency measure can be defined as follows:


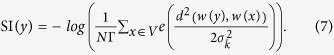


In our application, the intensity-based saliency finally will be normalized into range [0,1]. *d* is estimated by relative average distance. The relative distance is used in case the distribution of the data is not uniform, and the distance metric mainly focuses on the relationships between neighboring points. Let a patch set *W* in a vessel contains *n* patches *w*_1_,*w*_2_,...*w*_*n*_. The relative average distance of a pair of patches *w*(*x*), *w*(*y*) ∈ *W* is defined as follows:


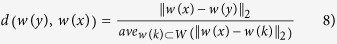


The 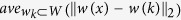 are the average Euclidean distance between *w*(*x*) and other patches *w*(*k*) belonging to *W* respectively. For two sets of points/pixels with similar neighboring relationships but different densities (i.e., similar relative density), the absolute distances between corresponding points differ dramatically from each other, but the relative distances are in general similar. This is an advantage of the relative distance in reflecting the relative density of points and relative scale of the objects.

### Shape-based Vessel Saliency

Let *u* be the diameter of *p*_1_ and *p*'_1_, and *v* be the diameter of *p*_2_ and *p*'_2_. Let *c*_1_ and *c*_2_ be the centre points of these two diameters, and their coordinates *c*_1_ = (***x***_*u*_, ***y***_*u*_) and *c*_2_ = (***x***_*v*_, ***y***_*v*_). Denotes *u*(*p*_1_, *p*'_1_), *v*(*p*_2_, *p*'_2_) are two random diameters of a given vessel, where (*p*_1_, *p*'_1_) and (*p*_2_, *p*'_2_) are the edge points on the vessel. The dissimilarity values of diameter *u* due to *v* in terms of length is given by *L* (*u*, *v*), where,


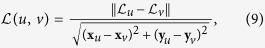


where 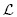 is diameter length, and 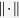 calculates the Euclidean distance of centre points of diameter of *u* and *v* in the vessel. The dissimilarity values of the orientation of each centreline pixels *c*_1_ and *c*_2_ is calculated as


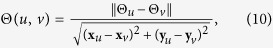


where Θ is the orientation of each pixel located on the centreline. After all the dissimilarity values in terms of diameter length and orientation, they are fused as weighted values 
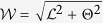
. A dissimilarity measure between a pair of diameters may be given as:


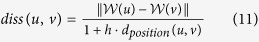


where *h* is the control parameter, and *h* = 3 in our implementation. Let *d*_*position*_(*u*, *v*) be the Euclidean distance between the centre points *c*_*u*_ and *c*_*v*_ of the two diameters of *u* and *v*.

We need to compute a distinctness value for each diameter, given the dissimilarity values calculated above. Diameter *u* is considered salient when it is highly dissimilar to other vertices, i.e., when *diss*(*u*, *v*) is high: ∀*v*. The saliency value of *u* is defined as


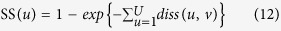


where *U* is the total number of the diameter in a given vessel. However, in practice, to evaluate the uniqueness of a diameters, there is no need to incorporate its dissimilarity to all the other diameters. If the most similar diameters (low dissimilarity diameters) are significantly different from diameter *u*, then clearly all diameters are also highly different from diameter *u*. Therefore, for diameter *u*, we search for the *M* most similar diameter according to the dissimilarity values, and define the diameters set as 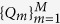. Hence, the saliency value of diameter *u* can be rewritten as


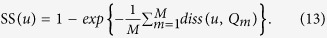


In practice, *M* is the number of diameters whose dissimilarity value are higher than the average dissimilarity value. Similarly, the shape-based saliency values are also normalized into [0,1].

**Figure 6:**
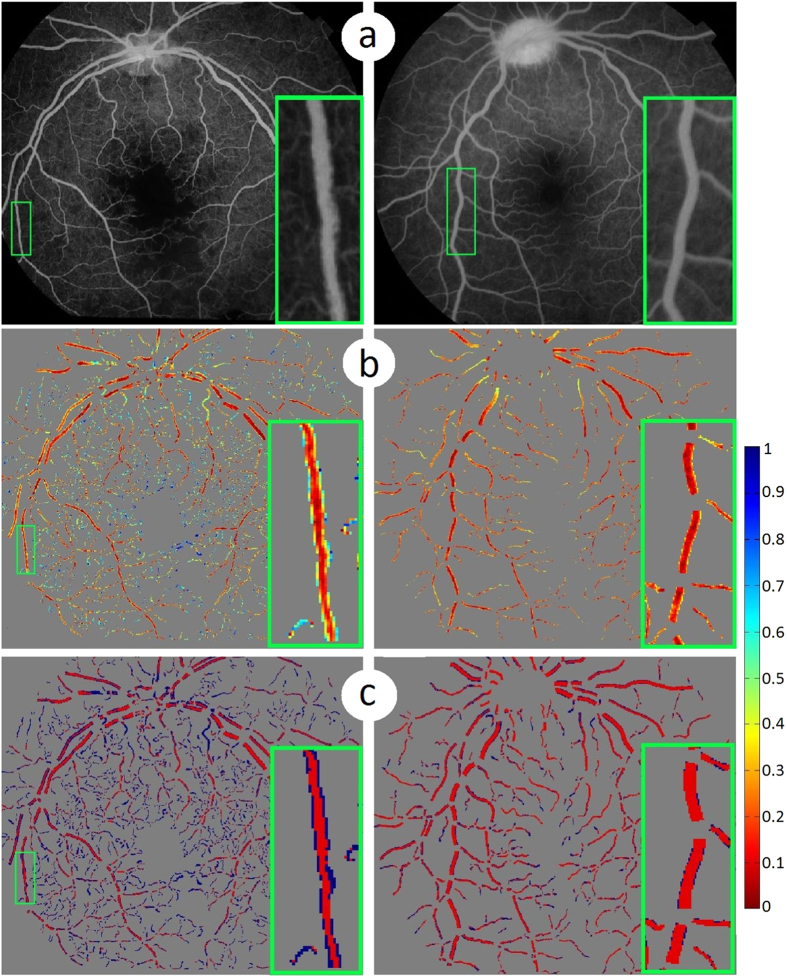
Illustration of saliency maps. (**a**) Two example fluorescein angiography images. (**b**) Saliency maps of each individual vessel segments. (**c**) Vessels are divided into salient and non-salient regions after applying thresholding process to images in the second row respectively. Blue colour indicates the most salient regions while red colour shows the least salient regions for images in the second and third rows. The inset in each image shows the zoom-in view of the region enclosed by the green box within that image.

After obtaining the saliency values for each pixel of vessel and vessel centreline, the shape-based saliency and intensity-based saliency are simply combined together into a final saliency map SM (SM = SI + SS), as shown as Fig. 6(b), the blue colour indicates the most salient regions, and the red colour shows the least salient regions. Two example images were selected: one with many abnormal vessel contained (top image of Fig. 6), and one with few abnormalities (bottom image of Fig. 6). It is clear that the salient regions in the top image are relatively more than the salient regions from the bottom one.

According to the pixel number of abnormal regions of each vessel in the final saliency map, the abnormality rate *R*(***v***) for each vessel is calculated as:


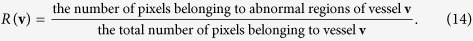


Figure 6(c) illustrates the saliency map after the thresholding process applied: the vessel regions where their saliency values is larger than an empirically defined threshold value of 0.65 will be set to 1 (abnormal), otherwise will be decided to 0 (normal).

### Feature Vector

Based on the saliency maps derived above, a feature vector of 21 features is derived for each vessel segment. These features are listed below:

#### Feature 1–4

Mean, standard deviation, entropy and the sum of gradient magnitude of the intensity-based saliency SI within the segment.

#### Feature 5–8

Mean, standard deviation, entropy and the sum of gradient magnitude of the shape-based saliency SS within the vessel segment.

#### Feature 9

Saliency-based abnormality rate *R* of the vessel segment.

#### Other Feature 10–21

Curvatures of the edges and the centerline of the vessel. Mean, standard deviation and entropy values of orientations of the two edges and the centerline.

### AdaBoost Classification

In this work we have used the AdaBoost classifier^47^ with weighted cost coefficient classifier for the purpose of classification task. AdaBoost works by building a stronger and more powerful classifier from lots of smaller weak classifiers. We used a decision tree as the weak classifier^47^. The weak classifiers are generated sequentially in order to decrease the estimation error of the previous weak classifier^48^. Although various classification techniques have been proposed, such as artificial neural networks, support vector machine (SVM), decision trees, the choice of classifier is dependent on the complexity of that specific application and the nature of the data. The reasons for our choice of weighted AdaBoost are three-fold. First, AdaBoost is relatively simple, easy to train and less susceptible to over-fitting than other classifiers. As such it usually provides relatively good performance for most classification problems^48^. Second, as an ensemble classifier it can be more effective than a single classifier in many cases, though this depends on the statistical properties of the data being analysed. Third, different weights can be easily introduced to tackle challenging classification problems. A weighted AdaBoost classifier has two parameters (class weights and number of trees) that have to be optimized in order to achieve the best classification performance. As described in Section Experiments, these are determined by the repeated LOOCV.

## Additional Information

**How to cite this article**: Zhao, Y. *et al.* Automated Detection of Vessel Abnormalities on Fluorescein Angiogram in Malarial Retinopathy. *Sci. Rep.* **5**, 11154; doi: 10.1038/srep11154 (2015).

## Figures and Tables

**Figure 1:**
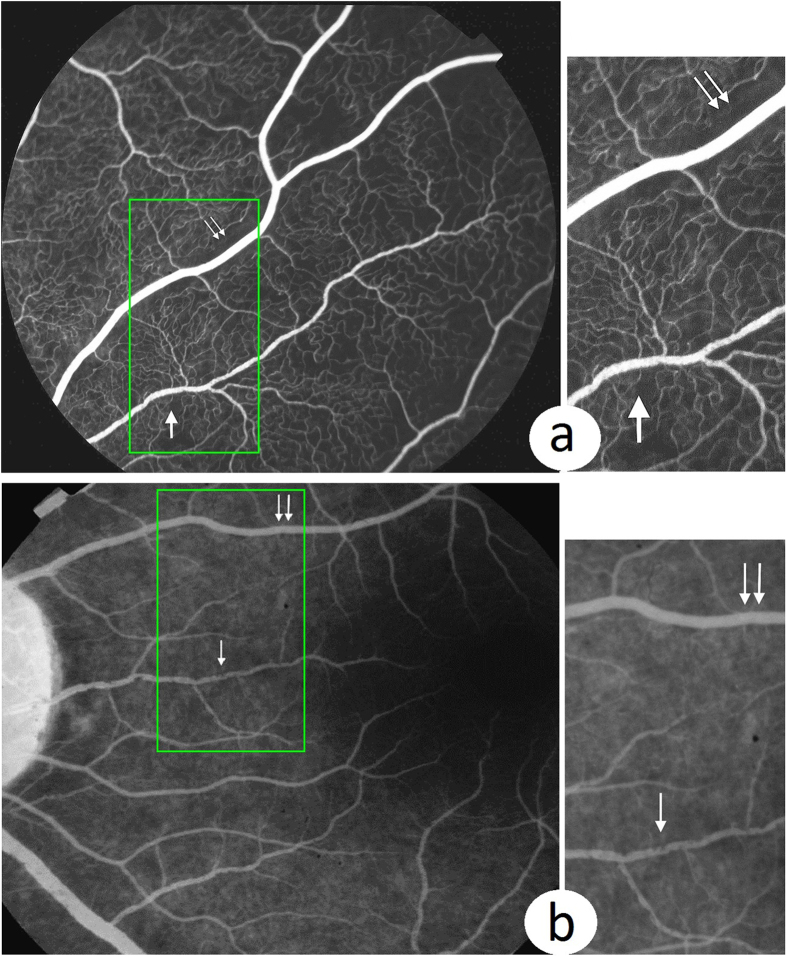
Two example fluorescein angiography images illustrating the appearances of IVFDs. Vessels with IVFD are shown by single arrows. Vessels without IVFD, in the same image, are shown by double arrows.(**a**) Example 1: Intensity of mature parasitized red blood cells in vessels with IVFD is significantly different from normal vessels. (**b**) Example 2: Edges of vessels with IVFDs become unsmooth, the diameter is changed dramatically when compared to normal vessels. The images on the right are the zoom-in view of the regions enclosed by the green box within the original image on the left respectively.

**Table 1:** Detection performance of the proposed framework for all the vessel segments, and for vessel segments with different types (large, small and peri-capillary vessels).

	**sensitivity**	**specificity**	**accuracy**	**AUC**
overall	0.747	0.735	0.741	0.742
large	0.732	0.713	0.729	0.728
small	0.765	0.782	0.751	0.776
peri-capillary	0.743	0.709	0.743	0.723
